# Age-Related Aspects of Sex Differences in Event-Related Brain Oscillatory Responses: A Turkish Study

**DOI:** 10.3390/brainsci14060567

**Published:** 2024-06-03

**Authors:** Görsev Yener, İlayda Kıyı, Seren Düzenli-Öztürk, Deniz Yerlikaya

**Affiliations:** 1 Faculty of Medicine, Izmir University of Economics, 35330 Balçova, Turkey; 2 Izmir Biomedicine and Genome Center, 35340 İzmir, Turkey; 3 Department of Neurosciences, Health Sciences Institute, Dokuz Eylül University, 35210 İzmir, Turkey; ilaydaky@gmail.com (İ.K.); psk.denizyerlikaya@gmail.com (D.Y.); 4 Department of Speech and Language Therapy, Faculty of Health Sciences, Izmir Bakırçay University, 35665 İzmir, Turkey; seren.duzenliozturk@bakircay.edu.tr

**Keywords:** oscillations, gender, sex, aging, EEG, event-related, task-related, ERP, P300, oddball

## Abstract

Earlier research has suggested gender differences in event-related potentials/oscillations (ERPs/EROs). Yet, the alteration in event-related oscillations (EROs) in the delta and theta frequency bands have not been explored between genders across the three age groups of adulthood, i.e., 18–50, 51–65, and >65 years. Data from 155 healthy elderly participants who underwent a neurological examination, comprehensive neuropsychological assessment (including attention, memory, executive function, language, and visuospatial skills), and magnetic resonance imaging (MRI) from past studies were used. The delta and theta ERO powers across the age groups and between genders were compared and correlational analyses among the ERO power, age, and neuropsychological tests were performed. The results indicated that females displayed higher theta ERO responses than males in the frontal, central, and parietal regions but not in the occipital location between 18 and 50 years of adulthood. The declining theta power of EROs in women reached that of men after the age of 50 while the theta ERO power was more stable across the age groups in men. Our results imply that the cohorts must be recruited at specified age ranges across genders, and clinical trials using neurophysiological biomarkers as an intervention endpoint should take gender into account in the future.

## 1. Introduction

Gender differences have profound societal implications as they influence brain development, behavior, and the presentation, prevalence, and treatment of diseases. However, women have historically been underrepresented in medical research [1]. This lack of representation can negatively impact women’s health outcomes. For example, from 1999 to 2000, 8 out of 10 drugs withdrawn from the market by the FDA posed higher risks for women than men [2]. More research is needed to better understand the neurobiology, behaviors, and disease vulnerabilities that may differ between sexes.

Research shows gender differences in the patterns of brain activity during memory retrieval tasks. Males tend to show greater activity than females in the prefrontal cortex, visual processing regions, parahippocampal cortex, and cerebellum [2,3], and the connectivity in brain networks associated with executive function and memory also differs between sexes and changes with age [4].

Overall, it is claimed that the brain metabolism in females is different from that in males throughout adulthood, which could impact vulnerability or resilience to neurodegenerative diseases [5]. The risk of developing AD is about twice as high for women compared to men globally [6]. However, the incidence rates appear to vary between low-to-middle-income countries, possibly due to several factors like survival rates between sexes, education levels, and genetic or hormonal influences [7]. Furthermore, increased female vulnerability to Alzheimer’s disease (AD) biomarkers in cerebrospinal fluid linked to greater hippocampal atrophy and faster age-related cognitive decline have been reported previously [8,9].

Neurophysiological signatures, measured with electroencephalography (EEG) or magnetoencephalography (MEG) techniques, have been stated as a low invasive and useful approach to investigating the progressive loss of neuronal activity in many cognitive impairments, including the Alzheimer’s disease (AD) continuum [10,11]. The early pioneering work of Başar et al. on task-related potentials has shown that event-related oscillations (EROs) provide a powerful technique, with a high temporal resolution, and can be used as a tool for detecting subtle abnormalities in cognitive processes [12,13]. While ERPs can be characterized by the amplitudes and latencies of the main wave components in the time domain, these wave components represent summed-up time-varying neural activation patterns of various neural circuits [12]. Thus, a greater understanding of the simultaneous involvement of several brain networks in brain activities has resulted from the analysis of the oscillatory dynamics of the ERP signal, also known as event-related oscillations (EROs) [13]. Delta and theta ERO responses are specifically important in the cognitive process and they are the main constituents of the ERP [14,15,16]. Gender is an important factor in event-related potentials/oscillations (ERPs/EROs), with higher amplitudes in females [17,18]. However, there have been no ERO studies related to gender effects in healthy populations across young, middle-aged, and older adults from low–middle-income countries such as Turkey in the previous literature.

In this vein, the purpose of this study was to assess and explore whether the amplitudes of event-related oscillations (EROs) in the delta and theta frequency bands change across age groups according to gender differences. As an outcome, it would be important to consider gender across various healthy older adult age groups over 50 years in a cohort of healthy individuals in Turkey.

## 2. Materials and Methods

### 2.1. Participants

For this study, the EEG data recorded from cognitively healthy participants at the Department of Neuroscience at the Health Sciences Institute of Dokuz Eylül University were scanned retrospectively. As a result of reviewing the database, data from 155 healthy participants who underwent a neurological examination, comprehensive neuropsychological assessment (including attention, memory, executive function, language, and visuospatial skills), and magnetic resonance imaging (MRI) in past studies were used.

The following standards were used to classify the participants as healthy: (1) no history of neurological or psychiatric disorders; (2) no significant vascular lesion load on their MRI as reviewed by an expert neurologist; (3) no self-reported cognitive complaints; and (4) neuropsychological test scores within the range of age–education–gender-adjusted norms.

We had six groups in this study: [3 (age: young, middle-aged, elderly) × 2 (gender: female and male)]. The demographic and clinical characteristics, neuropsychological profiles, and age characteristics of groups are reported in Table 1.

The following were used as as exclusion criteria: (1) depression scale scores above cut off (the Beck Depression Scale (BDS) for participants younger than 55 years and the Geriatric Depression Scale (GDS) for those older than 56 years), (2) neurological and psychiatric disease history, (3) usage of medication which may affect the cognitive skills, (4) vision problems which may affect the performance in the task during the EEG recording, (5) vascular lesions and/or atrophy on brain MRI, (6) alcohol and/or drug misuse, (7) history of head trauma, and (8) the mental counting performance with more than 10% error rate during the EEG recording. Consent forms were taken from all participants according to the Declaration of Helsinki.

### 2.2. Experimental Paradigm

In this study, EEG data were recorded during a visual oddball paradigm. All recordings with the paradigm were performed in an electrically isolated room during the morning hours. The total number of stimuli was 120 (40 target/80 nontarget). Visual stimuli were presented as light (for the target stimulus: 40 cd/m^2^; for the nontarget stimulus: 10 cd/m^2^) from a 22″ computer screen 120 cm in front of the participants with a refresh rate of 60 Hz in pseudorandom order. The paradigm consisted of 4 blocks and the inter-stimulus interval was varied by 3–7 s randomly. Participants were asked to do mental counting for the target stimulus. The participants were excluded with more than the 10% error rate (allowed range: 36–44).

### 2.3. EEG Recording and Data Processing

The EEG was recorded using 30 Ag/AgCl electrodes positioned on a standardized elastic cap according to the international 10–20 system (EasyCap; Brain Products GmbH; Gilching, Germany). The bilateral and linked earlobe electrodes (A1 + A2) were chosen as reference electrodes, and the electrooculogram (EOG) was recorded from the medial upper and lateral orbital rims of the right eye. All electrode impedances were kept below 10 kΩ. The EEG was amplified using a Brain Amp 32-channel DC amplifier with a 0.3–70 Hz band-pass filter and was digitized online with a sampling rate of 500 Hz.

Offline data preprocessing and analysis were performed using Brain Vision Analyzer 2.2 Software (Brain Products GmbH; Gilching, Germany). The 0.1 Hz high-pass filter with a zero-phase shift Butterworth filter and the 50 Hz Notch filter were applied to raw EEG data. Extended Infomax Independent Component Analysis (ICA) was used to correct horizontal and vertical eye movement artifacts. The target trials were segmented into 1500 ms epochs to include 500 ms pre-stimulus and 1000 ms post-stimulus activity. Automatic artifact rejection processing was performed with the following criteria: (a) maximum amplitude in an epoch: ±70 μV, (b) maximum allowed voltage step: 50 μV/ms, (c) maximum allowed difference in a 200 ms interval: 50 μV, (d) lowest activity in a 100 ms interval: 0.5 μV. The artifact-free epochs were averaged as time-locked to the stimulus onset for each participant. High- and low-pass filters with 8 dB/octave slope were applied to the averaged data to obtain the delta (0.5–3.5 Hz) and theta (4–7 Hz) oscillations. Frontal (F3, Fz, F4), Central (C3, Cz, C4), Parietal (P3, Pz, P4), and Occipital (O1, Oz, O2) electrodes were pooled. For each participant, the peak-to-peak activities of delta and theta oscillations were measured from the time range 0–800 ms for the delta and 0–500 ms for the theta after stimulus onset.

### 2.4. Statistical Analysis

Statistical analyses were carried out using IBM SPSS Statistics v. 24.0. Mixed-design ANOVA model with one 4-level within-subject factor (LOCATION: Frontal, Central, Parietal, Occipital) and two between-subject factors with 3 levels (AGE: Young, Middle-aged, Elderly) and 2 levels (GENDER: Female and Male) was performed to compare group differences for delta and theta amplitudes separately. Bonferroni correction was applied in post hoc analyses. The value of *p* < 0.05 was considered statistically significant for ANOVA analyses. Pearson’s correlation analysis was used to investigate the relation between age and the amplitude values of delta and theta oscillations for each gender separately. The value of *p* < 0.01 was considered statistically significant for correlation analyses.

## 3. Results

The EEG analyses included 155 healthy participants. All data were analyzed for delta and theta frequency bands separately using Mixed-design ANOVA.

### 3.1. Delta Frequency Band Results

In the delta band responses, there was no statistically significant main AGE [F(2, 149) = 2.369, *p* = 0.097], main GENDER [F(1, 149) = 0.022, *p* = 0.884], AGE × GENDER interaction effect [F(12, 149) = 1.073, *p* = 0.344], and AP × AGE × GENDER interaction effect [F(6, 447) = 1.146, *p* = 0.334].

### 3.2. Theta Frequency Band Results

**Figure 1:**
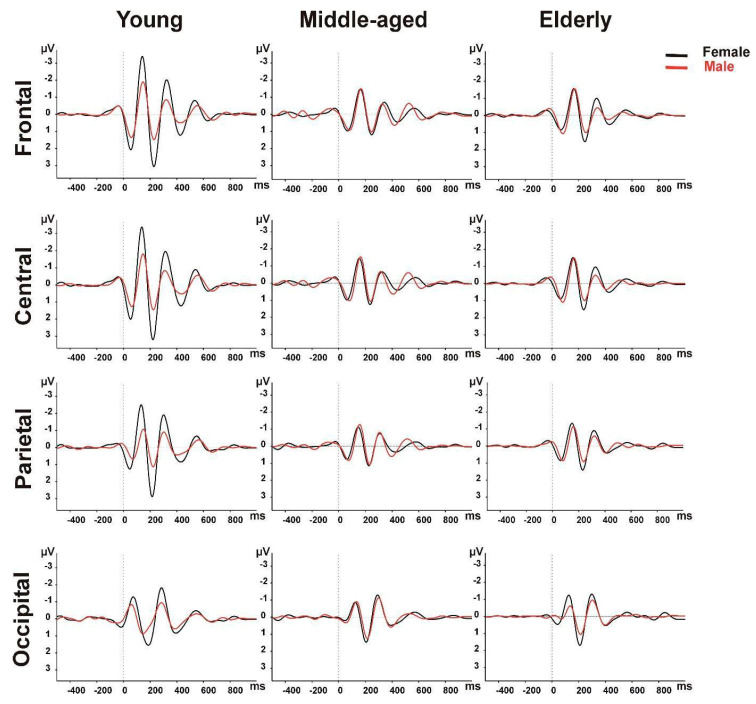
Grand averages of theta ERO power across age groups of both genders.

In the theta band responses, there is a significant AP × AGE × GENDER interaction effect [F(6, 447) = 5.276, *p* = 0.001]. In the post hoc analysis for the young adults, the female participants had greater theta responses than the males at the frontal, central, and parietal locations (all, *p* ≤ 0.001); at the occipital location, there was no significant gender difference (*p* = 0.178). For the elderly adults, at the occipital area, the females had a greater theta response than the males (*p* = 0.034), but at the other locations, there was no significant gender effect (all, *p* ≥ 0.272). In addition, for the middle-aged group, we did not find any significant gender effect for the theta responses (all, *p* ≥ 0.519) (Figure 1, Table 2).

In the pairwise comparisons, when we compared the topographical distributions among the age and gender groups, for the young group, we found that the females had showed the greatest responses at the frontal and central locations and the lowest response at the occipital location (all, *p* ≤ 0.019). On the other hand, the young males and all the participants in the middle-aged and elderly groups showed no locational significant difference (all, *p* ≥ 0.236).

### 3.3. Correlations

#### 3.3.1. Age

**Figure 2:**
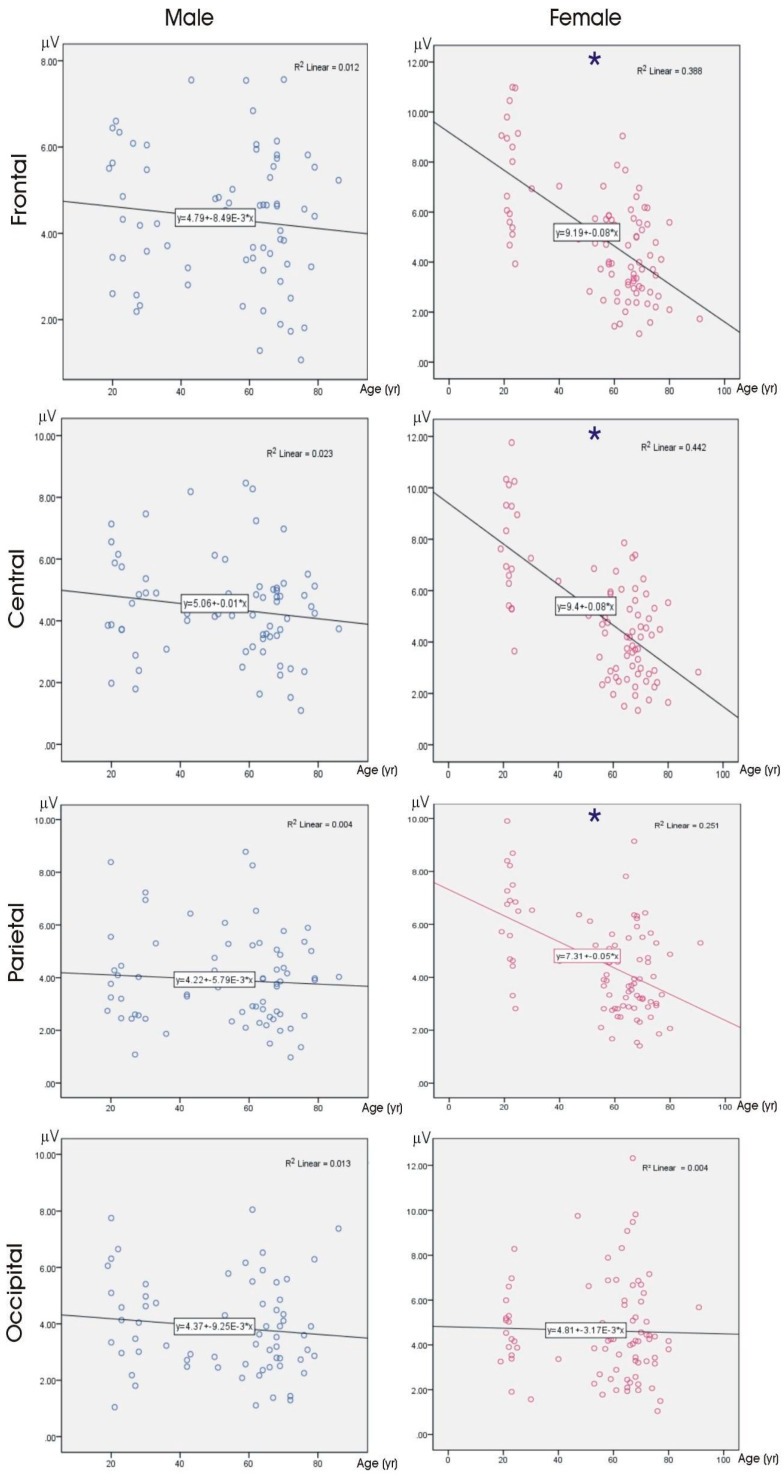
Correlational plots between theta ERO power and age in both genders.

For the female participants, negative and medium correlations between the theta ERO amplitudes and the age for the frontal, central, and parietal areas (all, *p* < 0.001) were noted. We found no significant correlations between the age and ERO amplitudes in the delta or theta frequency bands for the male participants and the delta frequency band for the female participants. The significant correlations are presented as scatter plots in Figure 2.

#### 3.3.2. Cognitive Functions

The correlation analyses showed significant moderate to strong negative correlations between the age and OVMPT total scores (r = −0.552, *p* < 0.001), OVMPT-IR (r = −0.383, *p* = 0.001), OVMPT-FR (r = −0.345, *p* = 0.004), digit span forward (r = −0.488, *p* < 0.001), and digit span backward (r = −0.502, *p* < 0.001) for the males. For the female participants, the correlation analysis revealed that there were moderate to strong negative correlations between the age and MMSE (r = −0.360, *p* = 0.001), OVMPT total scores (r = −0.501, *p* < 0.001), OVMPT-IR (r = −0.504, *p* = 0.001), semantic fluency (r = −0.312, *p* = 0.004), lexical fluency (r = −0.584, *p* < 0.001), digit span forward (r = −0.477, *p* < 0.001), and digit span backward (r = −0.478, *p* < 0.001). None of the correlations survived between EEG parameters and the neuropsychological tests when the statistical threshold was set at *p* < 0.01 to account for the inflating effects of testing.

## 4. Discussion

In the current study, we found that task-related brain responses change differently in females than males across adulthood. The three main findings of our study are as follows: (1) the higher theta EROs in females than males until middle age; (2) the declining theta power of ERO in women reaching that of men after the age groups of middle age years; and (3) stability of the theta ERO power is greater in men, in contrast with women’s declining theta power with age.

Mainly, in the current study, the females displayed higher theta ERO responses than the males until the middle age. These amplitude differences were observed in the frontal, central, and parietal regions but not in the occipital region between the genders between 18 and 50 years of adulthood.

The rsEEG and task-related EEG are widely studied for cognitive disorders in clinical research [19,20,21,22]. Among the task-related EEG methods, P300 is the most commonly used event-related potential (ERP) elicited after the application of a cognitive task, mostly the oddball paradigm [18,23]. Event-related oscillations (EROs) are elicited after the spectral analysis of ERPs and provide a powerful technique with a high temporal resolution, and it can be used as a tool for detecting subtle abnormalities in cognitive impairments or processes [12,24,25,26]. The dynamics of EROs are different than in rsEEG. In rsEEG, the increase in the delta and theta rhythms indicates a cognitive decline or pathology [19,20,27], whereas, in ERO, the same finding means a greater brain response to the task in the post-stimulus era [24]. In this vein, the cognitive performance is negatively associated with the rsEEG rhythms in the theta and delta frequencies [19,20,27]. In contrast, the ERO responses in the same frequency bands indicate a higher cognitive performance [25] and greater brain volume [22,28]. Regarding the functional role of delta ERO responses, Demiralp et al. (2001) reported that, after the application of stimuli with decreasing intensities, the oscillatory responses occur nearly in the pure delta range when stimuli reach closer to the subjective threshold [29]. At the threshold intensity, the stimulation focuses the attention of the subject. In this context, the lower delta oscillatory responses in older subjects upon either the visual or auditory oddball paradigm are quite understandable, as healthy elderly individuals tend to show decreased attentional or decision-making focus. In previous studies, regardless of modality, delta ERO studies have shown decreased amplitudes at the frontal and central locations in Alzheimer’s disease (AD) [30], Lewy body dementia (LBD), and Parkinson’s disease (PD) dementia [31]. This finding indicates supramodal alterations in the anterior parts of the hemispheres in various dementia patients, differentiating them from healthy controls [31]. As expected, smaller delta and theta ERO responses were associated with lower neuropsychological test scores in either auditory or visual modality in dementia groups [31], indicating a supramodal effect.

Previously, an auditory event-related oscillation study showed higher power of the theta and beta1 responses in females, yet the connectivity in these frequency bands was greater in males [32]. This finding was explained by the thinner calvarium in women, yet, another study found the cranium thickness is larger in females compared to males [33], which must lead to fully opposite results. The thicker cranium might lead to diminished EEG transmission from source to scalp electrode. Taking into consideration the fact that, on average, females have thicker cranium, the intracranial gender EEG power differences must be even stronger. Another ERO study [34] reported increased theta and alpha power in males in the age range of 20–29 years. This contradicting result might be more related to the age range of the sample than in our cohort. In a study similar to ours [35], studying the male and female developmental trajectories of theta ERO power, the genders were significantly different in their temporal characteristics, with more rapid decreases with age in males than in females during the ages of 12 to 25. The change in the rate of decrease with age was nearly monotonic in males, with greater fluctuations in females. The influence of gender on rsEEG rhythms during physiological aging was explored to understand neurophysiological mechanisms. Investigators found higher theta power density [36] and higher rsEEG amplitudes of the delta, theta, and alpha rhythms [37] in healthy females than males. Another recent retrospective and exploratory study on rsEEG indicated gender-related effects on the spatial frequency cortical sources of rsEEG rhythms in healthy controls and the mild cognitive impairment in Alzheimer’s disease (ADMCI) seniors, especially in the delta, alpha2, and alpha3 frequency bands [27].

This relationship between event-related brain oscillatory mechanisms in the delta and theta frequency bands and frontal executive functions may imply aberrant supramodal mechanisms and the impaired coordination of signal transmission [38] underlying the cognitive deficits in dementia patients [31]. Therefore, the higher theta ERO power with a declining slope in healthy women is a distinct pattern from that of healthy men during aging. This declining slope during aging may be related to the increased likelihood of women’s tendency to develop [1]. The mentioned studies suggested that the task-related oscillatory responses in the theta frequency band but not in the delta frequency band may provide evidence for aging-related effects earlier. Furthermore, our group’s earlier work on P300 ERP amplitudes [18], overlapping with the current study’s cohort, revealed greater P300 amplitudes in women, implying that the greater P300 amplitude in women may be driven by theta rather than delta ERO responses.

Regarding the functional correlates of the theta and delta ERO responses, previous studies reported that more pronounced ERO responses in the theta frequency range were induced during the cognitive processing of auditory stimuli [39], whereas the delta frequency range was induced during the cognitive processing of visual stimuli in healthy individuals [39,40]. This finding was explained by the longer distance for the synchronization of neural networks after visual stimuli and the shorter distance after auditory stimuli. In contrast to this notion, the results of the current study using visual stimuli indicate that the greater theta ERO in women cannot be explained solely based on distance.

Furthermore, the both delta [41,42,43,44,45] and theta ERO responses [41,42,46,47,48] are impaired in cognitively impaired patients due to AD, PD, and LBD. A study by Yener et al. (2019) in AD and PD mild cognitive impairment (MCI) patients reported that both groups displayed decreased theta ERO power; however, theta phase locking was impaired only in PDMCI patients [15]. The theta ERO phase locking abnormality in the PDMCI group in contrast to the ADMCI group was explained by a more vulnerable subcortical–thalamic loop in the PD patients.

The mentioned studies suggest that a higher theta ERO in women in our healthy, relatively young participants up to the age of 50 implies greater or more effortful processing during a cognitive task [15,49]. This may be part of the subcortical ascending systems modulating the thalamocortical loop and generating ERO delta–theta responses [50]. Therefore, it can be hypothesized that EROs in slow frequency bands, i.e., delta and theta, may modulate the efficiency of neural transfer supporting various aspects of cognitive abilities including attention, executive functions, decision-making, and memory [12,51], and may regulate the cortico-striatal and thalamocortical re-entrant signals to the cerebral cortex during the processing of cognitive stimuli [31]. Within this context, the greater augmentation in the ERO theta responses to the oddball target stimuli may be associated with more integration between the prefrontal–cortical and subcortical networks in younger women. However, this augmented response disappears with a declining slope until the age of 50 in women, becoming similar to that of men.

The explanations for these distinct ERO profiles of the two genders mentioned here can be threefold: firstly, Alzheimer’s disease (AD) is a greater threat for older women than men [52], and with age, the EEG patterns in elderly individuals become more similar to those of individuals in the early stages of the AD continuum [20,53]. This finding could be due to the accumulation of pathological peptides many years before any clinical manifestation [54]. In our study, in women, the decreasing theta ERO power with age may be a reflection of the increased risk of developing a neurodegenerative disorder in relation to gender. Since the AD biomarkers were not included in the current study, we cannot be sure if some individuals in our cognitively healthy elderly cohort have started accumulating amyloid in their brains, since it is a well-known fact that characteristic pathological peptide accumulation starts many years before any clinical presentation of AD [54]. Furthermore, several studies have reported gender differences regarding the progression of the neuropathological and clinical manifestations of AD [55,56]. AD disproportionally affects females; females have greater total brain atrophy, display a greater incidence rate, and perform worse in many neuropsychological tasks [57]. Secondly, these results could be related to the longer life expectancy of women in comparison to men [7]. The increased female vulnerability to cerebrospinal fluid (CSF) AD biomarkers is linked to greater hippocampal atrophy and faster age-related cognitive decline [8,9]. On the other hand, a report investigating gender-related EEG/CSF patterns in AD has shown altered rsEEG theta rhythms related to Ab-42 in men [58]. At this point, it is important to remember the different dynamics of rsEEGs and EROs in the theta frequency, where cognitive performance is negatively associated with theta rsEEG rhythms [59], whilst theta ERO responses are indicative of higher cognitive performance [16] and brain volume [22,45]. Thirdly, human brains show great plasticity during their lifespan [60], and education, gender, and sex hormones have an impact on brain plasticity [1,61]. Female-specific genes were reported in a meta-analysis on the gender effects of AD gene expression, where these genes were involved in pathways such as oxidative phosphorylation and were associated with neurodegenerative diseases [62].

Regarding the distinctively different associations between age and neuropsychological test patterns across genders, in our sample, the women tended to decline in verbal fluency while men declined in memory tests. The previous literature supports our findings. The decline in memory and visuospatial skills varied across ethnic groups, with black women displaying sharper declines in memory and visuospatial abilities than Hispanic males and non-Hispanic white women, respectively [63]. Additionally, the cognitive traits were reported to be different between genders; for example, navigation by mental maps, mental rotation, visuo-spatial skills, and working memory were stronger in men, while navigation by landmarks, verbal ability, reading and writing abilities, fine motor coordination, and perceptual skills higher in women [1]. Brain health in diverse settings, including geographical differences, age, cognition, and demographics, can affect EEG-related changes [64,65,66,67]. This study provides insights on the neuropsychological test profile between genders in a sample of the Turkish population in the West Asia region.

The current study did not display the association of the theta or delta ERO responses with cognitive tests. This finding is not in line with our expectations, as previous studies indicated theta and delta EROs are correlated with cognitive functions [31]. The reason for this discrepancy may be due to the size and the age profile of our group, and secondly, to the blunting effect of different cognitive decline patterns across genders. Another reason for the lack of correlation between neuropsychological testing and EEG parameters could be the absence of significant cognitive impairments in the study participants.

Briefly, the findings of the current exploratory study support the hypothesis that gender-related changes may be important when establishing normative data in EROs for both clinical and research applications. The expansive data offered by new technologies render new opportunities for gender-stratified clinical decision support systems available [68]. Similarly, the development of data analysis approaches integrating clinical and demographic factors, including gender and aging, is crucial, especially for electrophysiological methodologies. For performing a gender-stratified and gender-adjusted analysis of all the EEG/ERO data, the next step should be the establishment and improvement of longitudinal cohorts with repeated assessments of clinical, cognitive, and biomarker variables, such as peripheral (plasma, neuroimaging, and cerebrospinal fluid), multi-omics (proteome, and metabolome), and genetic data [69]. EEG methods show high stability across the lifespan [70] and the internal consistency of task-related oscillations in the delta and theta frequency bands is very high, with absolute Cronbach’s alpha values of 0.90 and 0.94, respectively, indicating slow ERO responses are stable measurements for cognition in healthy adults [71]. Therefore, the results of our study indicate that the investigation of EROs should consider gender factors across different age groups.

The limitations of the current study include the lack of sexual hormone level data from the cohort and the relatively small number of participants for investigating gender effects. Although our healthy volunteers were neurologically intact and displayed normal limits of scores in the extensive neuropsychological tests and depression scales with no abnormality in their MRI images, they were not tested for CSF or plasma AD biomarkers. Additionally, the relatively less number of female participants in the range of 25–45 years might have accentuated the gender difference in the current paper. In future studies, adding datasets from cognitively unimpaired females of the mentioned age range and individuals with cognitive impairment to the sample might empower the statistical correlation model. Furthermore, the cohorts need to be recruited at specified age ranges, from women at pre- and post-menopausal ages to age-matched men. Then, EEG methodologies will have a continuous improvement in the diagnosis and therapy of disease.

In conclusion, in clinical trials using EEG biomarkers as an intervention endpoint, gender should be taken into account and EEG/ERO data should be enriched for performing gender-stratified analysis.

## Figures and Tables

**Table 1:** Demographic and clinical characteristics of participants.

			Age Groups			
	Total Sample	Gender	19–50 (n = 45)	51–65 (n = 48)	66–86 (n = 62)	*p* Values
Age (yr) *	55.01 ± 19.41	Female	24.80 ± 6.85	59.28 ± 3.88	71.32 ± 5.00	0.197
		Male	29.32 ± 9.46	60.16 ± 4.18	71.84 ± 5.07	
**Gender** **^‡^**	155 (86F/69M)	Female	20	29	37	0.209
		Male	25	19	25	
Education (yr) *	12.75 ± 4.37	Female	14.90 ± 1.71	12.45 ± 3.98	9.65 ± 5.11	0.005
		Male	14.79 ± 1.50	12.63 ± 3.13	14.08 ± 5.26	
**Handedness** **^‡^**	149 R/4L/2B	Female	20R	29R	36R/1L	0.126
		Male	24 R/1L	17R/2B	23R/2L	
Epoch Number *	28.75 ± 7.32	Female	26.10 ± 8.22	27.86 ± 6.88	28.14 ± 7.03	0.604
		Male	28.16 ± 6.25	32.63 ± 7.38	30.48 ± 7.60	
Behavioral Data *	39.69 ± 2.20	Female	40.40 ± 1.50	39.48 ± 2.03	39.49 ± 2.91	0.124
		Male	39.24 ± 2.50	39.79 ± 1.58	40.04 ± 1.70	
MMSE *	29.22 ± 1.04	Female	29.75 ± 0.55	28.93 ± 1.28	28.86 ± 1.12	0.592
		Male	29.70 ± 0.70	29.26 ± 0.99	29.16 ± 0.94	
**Depression** ^¥^	4.89 ± 3.69	Female	4.89 ± 3.70	9.00 ± 6.23	7.36 ± 5.00	NA
	5.54 ± 4.51	Male	5.58 ± 4.47	4.72 ± 4.40	4.20 ± 3.60	
OVMPT Total *	121.80 ± 12.88	Female	128.33 ± 9.24	122.38 ± 10.19	118.47 ± 12.45	0.467
	120.33 ± 14.79	Male	130.25 ± 9.31	120.64 ± 13.62	113.00 ± 14.70	
OVMPT IR *	6.23 ± 1.97	Female	7.42 ± 1.83	6.42 ± 1.58	5.25 ± 1.77	0.310
	5.99 ± 1.92	Male	7.42 ± 1.83	6.29 ± 2.02	5.25 ± 1.77	
OVMPT FR *	13.62 ± 1.20	Female	14.00 ± 1.10	13.65 ± 1.20	13.30 ± 1.29	0.456
	13.22 ± 1.43	Male	13.75 ± 1.06	13.21 ± 1.31	12.81 ± 133	
OVMPT TR *	14.99 ± 0.11	Female	15	15	15	0.995
	15.00 ± 0.00	Male	15	15	15	
Stroop *	48.09 ± 19.84	Female	31.17 ± 5.85	45.46 ± 14.80	53.00 ± 20.49	0.326
	43.12 ± 18.65	Male	40.50 ± 16.31	40.57 ± 12.33	49.63 ± 26.46	
Categorical Fluency *	22.81 ± 4.95	Female	25.00 ± 5.02	23.69 ± 5.36	22.48 ± 4.67	0.419
	24.27 ± 4.71	Male	26.42 ± 5.14	23.93 ± 4.73	24.63 ± 5.18	
Phonemic Fluency *	44.46 ± 14.09	Female	61.17 ± 9.47	40.42 ± 11.38	38.30 ± 12.04	0.007
	44.80 ± 12.79	Male	41.50 ± 11.94	48.50 ± 16.18	41.00 ± 10.68	
BNT *	14.79 ± 0.47	Female	15	14.96 ± 0.20	14.78 ± 0.42	0.716
	14.94 ± 0.31	Male	15	15	14.81 ± 0.54	

MMSE: The Mini Mental State Test, OVMPT: Öktem’s Verbal Memory Performance Test, IR: Immediate Recall, FR: Free Recall, TR: Total Recognition, BNT: Boston Naming Test. **^¥^** Depression Scores are according to Beck Depression Scale for young group and to Yesavage’s Geriatric Depression Scale for middle-aged and elderly group. * Univariate ANOVA; **^‡^** chi-squared test; NA: not applicable as scores were elicited by different tests.

**Table 2:** Amplitude values (μV) of EROs in theta frequency band according to age groups and gender.

		Female (X^−^ ± SD)	Male (X^−^ ± SD)	*p* Values
**Young** **(18–50 yr)**	Frontal	7.41 ± 2.22	4.49 ± 1.52	**<0.001**
	Central	7.56 ± 2.15	4.70 ± 1.69	**<0.001**
	Parietal	6.28 ± 1.83	4.03 ± 1.80	**<0.001**
	Occipital	4.80 ± 1.67	3.55 ± 1.41	0.178
**Middle-Aged** **(51–65 yr)**	Frontal	4.42 ± 1.95	4.34 ± 1.59	0.875
	Central	4.35 ± 1.64	4.52 ± 1.86	0.735
	Parietal	3.94 ± 1.37	4.23 ± 2.04	0.552
	Occipital	4.48 ± 2.02	4.10 ± 1.83	0.519
**Elderly** **(>65 yr)**	Frontal	3.94 ± 1.54	4.19 ± 1.62	0.581
	Central	3.97 ± 1.57	4.01 ± 1.37	0.940
	Parietal	4.03 ± 1.67	3.55 ± 1.41	0.272
	Occipital	4.65 ± 2.36	3.58 ± 1.51	**0.034**

## Data Availability

The data supporting the findings of this study are available through the corresponding author, upon reasonable request.

## References

[1] Zelco A., Wapeesittipan P., Joshi A. (2023). Insights into Sex and Gender Differences in Brain and Psychopathologies Using Big Data. Life.

[2] Harkin T., Snowe O.J., Mikulski B.A., Waxman H.A. (2001). Drug Safety: Most Drugs Withdrawn in Recent Years Had Greater Health Risks for Women.

[3] Spets D.S., Slotnick S.D. (2021). Are there sex differences in brain activity during long-term memory? A systematic review and fMRI activation likelihood estimation meta-analysis. Cogn. Neurosci..

[4] Malpetti M., Ballarini T., Presotto L., Garibotto V., Tettamanti M., Perani D. (2017). Alzheimer’s Disease Neuroimaging Initiative (ADNI) Database Network for Efficiency and Standardization of Dementia Diagnosis (NEST-DD) database. Gender differences in healthy aging and Alzheimer’s Dementia: A 18 F-FDG-PET study of brain and cognitive reserve. Hum. Brain Mapp..

[5] Goyal M.S., Vlassenko A.G., Raichle M.E. (2019). Reply to Biskup et al. and Tu et al.: Sex differences in metabolic brain aging. Proc. Natl. Acad. Sci. USA.

[6] Perera G., Pedersen L., Ansel D., Alexander M., Arrighi H.M., Avillach P., Foskett N., Gini R., Gordon M.F., Gungabissoon U. (2018). Dementia prevalence and incidence in a federation of European Electronic Health Record databases: The European Medical Informatics Framework resource. Alzheimers Dement..

[7] Masters C.L., Bateman R., Blennow K., Rowe C.C., Sperling R.A., Cummings J.L. (2015). Alzheimer’s disease. Nat. Rev. Dis. Primers.

[8] Koran M.E.I., Wagener M., Hohman T.J. (2017). Alzheimer’s Neuroimaging Initiative. Sex differences in the association between AD biomarkers and cognitive decline. Brain Imaging Behav..

[9] Li R., Singh M. (2014). Sex differences in cognitive impairment and Alzheimer’s disease. Front. Neuroendocrinol..

[10] Babiloni C., Triggiani A.I., Lizio R., Cordone S., Tattoli G., Bevilacqua V., Soricelli A., Ferri R., Nobili F., Gesualdo L. (2016). Classification of Single Normal and Alzheimer’s Disease Individuals from Cortical Sources of Resting State EEG Rhythms. Front. Neurosci..

[11] Maestú F., Cuesta P., Hasan O., Fernandéz A., Funke M., Schulz P.E. (2019). The Importance of the Validation of M/EEG With Current Biomarkers in Alzheimer’s Disease. Front. Hum. Neurosci..

[12] Başar-Eroglu C., Basar E., Demiralp T., Schürmann M. (1992). P300-response: Possible psychophysiological correlates in delta and theta frequency channels. A review. Int. J. Psychophysiol..

[13] Başar E., Karakaş S. (1998). Event-Related Oscillations in the Brain. Brain Function and Oscillations.

[14] Başar-Eroglu C., Demiralp T. (2001). Event-related theta oscillations: An integrative and comparative approach in the human and animal brain. Int. J. Psychophysiol..

[15] Yener G.G., Fide E., Özbek Y., Emek-Savaş D.D., Aktürk T., Çakmur R., Güntekin B. (2019). The difference of mild cognitive impairment in Parkinson’s disease from amnestic mild cognitive impairment: Deeper power decrement and no phase-locking in visual event-related responses. Int. J. Psychophysiol..

[16] Aktürk T., de Graaf T.A., Erdal F., Sack A.T., Güntekin B. (2022). Oscillatory delta and theta frequencies differentially support multiple items encoding to optimize memory performance during the digit span task. Neuroimage.

[17] Güntekin B., Başar E. (2007). Brain oscillations are highly influenced by gender differences. Int. J. Psychophysiol..

[18] Yerlikaya D., Hünerli-Gündüz D., Fide E., Özbek Y., Kıyı İ., Öztura İ., Yener G.G. (2022). The reliability of P300 and the influence of age; gender and education variables in a 50 years and older normative sample. Int. J. Psychophysiol..

[19] Babiloni C., Blinowska K., Bonanni L., Cichocki A., De Haan W., Del Percio C., Dubois B., Escudero J., Fernández A., Frisoni G. (2020). What electrophysiology tells us about Alzheimer’s disease: A window into the synchronization and connectivity of brain neurons. Neurobiol. Aging..

[20] Babiloni C., Lopez S., Noce G., Ferri R., Panerai S., Catania V., Soricelli A., Salvatore M., Nobili F., Arnaldi D. (2022). Resting State Alpha Electroencephalographic Rhythms Are Affected by Sex in Cognitively Unimpaired Seniors and Patients with Alzheimer’s Disease and Amnesic Mild Cognitive Impairment: A Retrospective and Exploratory Study. Cereb. Cortex..

[21] Yener G.G., Güntekin B., Örken D.N., Tülay E., Forta H., Başar E. (2012). Auditory delta event-related oscillatory responses are decreased in Alzheimer’s disease. Behav. Neurol..

[22] Yener G.G., Emek-Savaş D.D., Lizio R., Çavuşoğlu B., Carducci F., Ada E., Güntekin B., Babiloni C.C., Başar E. (2016). Frontal delta event-related oscillations relate to frontal volume in mild cognitive impairment and healthy controls. Int. J. Psychophysiol..

[23] Polich J., Kok A. (1995). Cognitive and biological determinants of P300: An integrative review. Biol. Psychol..

[24] Başar E., Başar-Eroğlu C., Güntekin B., Yener G.G. (2013). Brain’s alpha, beta, gamma, delta, and theta oscillations in neuropsychiatric diseases: Proposal for biomarker strategies. Suppl. Clin. Neurophysiol..

[25] Güntekin B., O’Donnell B.F. (2023). Special Issue: Update on Neural Oscillations in Neuropsychiatric Disorders. Clin. EEG Neurosci..

[26] Yener G.G., Emek-Savaş D.D., Güntekin B., Başar E. (2014). The visual cognitive network, but not the visual sensory network, is affected in amnestic mild cognitive impairment: A study of brain oscillatory responses. Brain Res..

[27] Babiloni C., Jakhar D., Tucci F., Del Percio C., Lopez S., Soricelli A., Salvatore M., Ferri R., Catania V., Massa F. (2024). Resting state electroencephalographic alpha rhythms are sensitive to Alzheimer’s disease mild cognitive impairment progression at a 6-month follow-up. Neurobiol. Aging..

[28] Hünerli D., Emek-Savaş D.D., Çavuşoğlu B., Dönmez Çolakoğlu B., Ada E., Yener G.G. (2019). Mild cognitive impairment in Parkinson’s disease is associated with decreased P300 amplitude and reduced putamen volume. Clin. Neurophysiol..

[29] Demiralp T., Ademoglu A., Istefanopulos Y., Başar-Eroglu C., Başar E. (2001). Wavelet analysis of oddball P300. Int. J. Psychophysiol..

[30] Yener G., Hünerli-Gündüz D., Yıldırım E., Aktürk T., Başar-Eroğlu C., Bonanni L., Del Percio C., Farina F., Ferri R., Güntekin B. (2022). Treatment effects on event-related EEG potentials and oscillations in Alzheimer’s disease. Int. J. Psychophysiol..

[31] Yıldırım E., Hanoğlu L., Yener G., Yerlikaya D., Taylor J.P., Schumacher J., McKeith I., Bonanni L., Pantano P., Piervincenzi C. (2023). Relationship between default mode network and resting-state electroencephalographic alpha rhythms in cognitively unimpaired seniors and patients with dementia due to Alzheimer’s disease. Cereb. Cortex..

[32] Georgiev S., Minchev Z., Christova C., Philipova D. (2011). Gender event-related brain oscillatory differences in normal elderly population EEG. Int. J. Bioautomation.

[33] Li H., Ruan J., Xie Z., Wang H., Liu W. (2007). Investigation of the critical geometric characteristics of living human skulls utilising medical image analysis techniques. Int. J. Veh. Saf..

[34] Langrova J., Kremláček J., Kuba M., Kubova Z., Szanyi J. (2012). Gender impact on electrophysiological activity of the brain. Physiol. Res..

[35] Chorlian D.B., Rangaswamy M., Manz N., Kamarajan C., Pandey A.K., Edenberg H., Kuperman S., Porjesz B. (2015). Gender modulates the development of theta event related oscillations in adolescents and young adults. Behav. Brain Res..

[36] Duffy F.H., McAnulty G.B., Albert M.S. (1993). The pattern of age-related differences in electrophysiological activity of healthy males and females. Neurobiol. Aging.

[37] Wada Y., Takizawa Y., Jiang Z.Y., Yamaguchi N. (1994). Gender differences in quantitative EEG at rest and during photic stimulation in normal young adults. Clin. Electroencephalogr..

[38] Spagna A., Wu T., Kim K., Fan J. (2020). Supramodal executive control of attention: Evidence from unimodal and crossmodal dual conflict effects. Cortex.

[39] Yordanova J., Kolev V., Rosso O.A., Schürmann M., Sakowitz O.W., Ozgören M., Basar E. (2002). Wavelet entropy analysis of event-related potentials indicates modality-independent theta dominance. J. Neurosci. Methods..

[40] Schürmann M., Başar-Eroglu C., Kolev V., Başar E. (2001). Delta responses and cognitive processing: Single-trial evaluations of human visual P300. Int. J. Psychophysiol..

[41] Yener G.G., Güntekin B., Oniz A., Başar E. (2007). Increased frontal phase-locking of event-related theta oscillations in Alzheimer patients treated with cholinesterase inhibitors. Int. J. Psychophysiol..

[42] Güntekin B., Aktürk T., Arakaki X., Bonanni L., Del Percio C., Edelmayer R., Farina F., Ferri R., Hanoğlu L., Kumar S. (2022). Are there consistent abnormalities in event-related EEG oscillations in patients with Alzheimer’s disease compared to other diseases belonging to dementia?. Psychophysiology..

[43] Rosenblum Y., Shiner T., Bregman N., Fahoum F., Giladi N., Maidan I., Mirelman A. (2022). Event-related oscillations differentiate between cognitive; motor and visual impairments. J. Neurol..

[44] Tülay E.E., Güntekin B., Yener G., Bayram A., Başar-Eroğlu C., Demiralp T. (2020). Evoked and induced EEG oscillations to visual targets reveal a differential pattern of change along the spectrum of cognitive decline in Alzheimer’s Disease. Int. J. Psychophysiol..

[45] Hünerli-Gündüz D., Özbek İşbitiren Y., Uzunlar H., Çavuşoğlu B., Çolakoğlu B.D., Ada E., Güntekin B., Yener G.G. (2023). Reduced power and phase-locking values were accompanied by thalamus; putamen; and hippocampus atrophy in Parkinson’s disease with mild cognitive impairment: An event-related oscillation study. Neurobiol. Aging..

[46] Missonnier P., Gold G., Herrmann F.R., Fazio-Costa L., Michel J.P., Deiber M.P., Michon A., Giannakopoulos P. (2006). Decreased theta event-related synchronization during working memory activation is associated with progressive mild cognitive impairment. Dement. Geriatr. Cogn. Disord..

[47] Rosenblum Y., Maidan I., Fahoum F., Giladi N., Bregman N., Shiner T., Mirelman A. (2020). Differential changes in visual and auditory event-related oscillations in dementia with Lewy bodies. Clin. Neurophysiol..

[48] Schmiedt C., Meistrowitz A., Schwendemann G., Herrmann M., Basar-Eroglu C. (2005). Theta and alpha oscillations reflect differences in memory strategy and visual discrimination performance in patients with Parkinson’s disease. Neurosci. Lett..

[49] Başar E., Güntekin B., Yener G., Başar-Eroğlu C. (2016). Mindful brain and EEG-neurophysiology. Int. J. Psychophysiol..

[50] Pfurtscheller G., Lopes da Silva F.H. (1999). Event-related EEG/MEG synchronization and desynchronization: Basic principles. Clin. Neurophysiol..

[51] Harmony T., Fernández T., Silva J., Bernal J., Díaz-Comas L., Reyes A., Marosi E., Rodríguez M., Rodríguez M. (1996). EEG delta activity: An indicator of attention to internal processing during performance of mental tasks. Int. J. Psychophysiol..

[52] (2021). Global Status Report on the Public Health Response to Dementia.

[53] Emek-Savaş D.D., Özmüş G., Güntekin B., Dönmez Çolakoğlu B., Çakmur R., Başar E., Yener G.G. (2017). Decrease of Delta Oscillatory Responses in Cognitively Normal Parkinson’s Disease. Clin. EEG Neurosci..

[54] Moscoso A., Grothe M.J., Ashton N.J., Karikari T.K., Lantero Rodríguez J., Snellman A., Suárez-Calvet M., Blennow K., Zetterberg H., Schöll M. (2021). Longitudinal Associations of Blood Phosphorylated Tau181 and Neurofilament Light Chain with Neurodegeneration in Alzheimer Disease. JAMA Neurol..

[55] Buckley R.F., Mormino E.C., Amariglio R.E., Properzi M.J., Rabin J.S., Lim Y.Y., Papp K.V., Jacobs H.I.L., Burnham S., Hanseeuw B.J. (2018). Sex, amyloid, and APOE e4 and risk of cognitive decline in preclinical Alzheimer’s disease: Findings from three well-characterized cohorts. Alzheimers Dement..

[56] Caldwell J.Z.K., Zhuang X., Leavitt M.J., Banks S.J., Cummings J., Cordes D., Papp K.V., Jacobs H.I.L., Burnham S., Hanseeuw B.J. (2019). Sex moderates amyloid and apolipoprotein e4 effects on default mode network connectivity at rest. Front. Neurol..

[57] Williamson J.N., James S.A., Mullen S.P., Sutton B.P., Wszalek T., Mulyana B., Mukli P., Yabluchanskiy A. (2024). Alzheimer’s Disease Neuroimaging Initiative Consortium; Yang, Y. Sex differences in interacting genetic and functional connectivity biomarkers in Alzheimer’s disease. GeroScience.

[58] Chino-Vilca B., Rodríguez-Rojo I.C., Torres-Simón L., Cuesta P., Vendrell A.C., Piñol-Ripoll G., Huerto R., Tahan N., Maestú F. (2022). Sex specific EEG signatures associated with cerebrospinal fluid biomarkers in mild cognitive impairment. Clin. Neurophysiol..

[59] Babiloni C., Arakaki X., Azami H., Bennys K., Blinowska K., Bonanni L., Bujan A., Carrillo M.C., Cichocki A., de Frutos-Lucas J. (2021). Measures of resting state EEG rhythms for clinical trials in Alzheimer’s disease: Recommendations of an expert panel. Alzheimers Dement..

[60] Maguire E.A., Gadian D.G., Johnsrude I.S., Good C.D., Ashburner J., Frackowiak R.S., Frith C.D. (2000). Navigation-related structural change in the hippocampi of taxi drivers. Proc. Natl. Acad. Sci. USA.

[61] Hirnstein M., Hausmann M. (2021). Sex/gender differences in the brain are not trivial—A commentary on Eliot et al. (2021). Neurosci. Biobehav. Rev..

[62] Moradifard S., Hoseinbeyki M., Ganji S.M., Minuchehr Z. (2018). Analysis of microRNA and Gene Expression Profiles in Alzheimer’s Disease: A Meta-Analysis Approach. Sci. Rep..

[63] Avila J.F., Vonk J.M.J., Verney S.P., Witkiewitz K., Arce Rentería M., Schupf N., Mayeux R., Manly J.J. (2019). Sex/gender differences in cognitive trajectories vary as a function of race/ethnicity. Alzheimer’s Dement..

[64] Hernandez H., Baez S., Medel V., Moguilner S., Cuadros J., Santamaria-Garcia H., Tagliazucchi E., Valdes-Sosa P.A., Lopera F., OchoaGómez J.F. (2024). Brain health in diverse settings: How age, demographics and cognition shape brain function. Neuroimage.

[65] Wang K., Umla-Runge K., Hofmann J., Ferdinand N.K., Chan R.C. (2014). Cultural differences in sensitivity to the relationship between objects and contexts: Evidence from P3. Neuroreport.

[66] Sonke C.J., Van Boxtel G.J., Griesel D.R., Poortinga Y.H. (2008). Brain wave concomitants of cross-cultural differences in scores on simple cognitive tasks. J. Cross-Cult. Psychol..

[67] Lewis R.S., Goto S.G., Kong L.L. (2008). Culture and context: East Asian American and European American differences in P3 event-related potentials and self-construal. Personal. Soc. Psychol. Bull..

[68] Kimenai D.M., Janssen E.B.N.J., Eggers K.M., Lindahl B., den Ruijter H.M., Bekers O., Appelman Y., Meex S.J.R. (2018). Sex-Specific versus Overall Clinical Decision Limits for Cardiac Troponin I and T for the Diagnosis of Acute Myocardial Infarction: A Systematic Review. Clin. Chem..

[69] Prado P., Birba A., Cruzat J., Santamaría-García H., Parra M., Moguilner S., Tagliazucchi E., Ibáñez A. (2022). Dementia ConnEEGtome: Towards multicentric harmonization of EEG connectivity in neurodegeneration. Int. J. Psychophysiol..

[70] Lopez K.L., Monachino A.D., Vincent K.M., Peck F.C., Gabard-Durnam L.J. (2023). Stability; change; and reliable individual differences in electroencephalography measures: A lifespan perspective on progress and opportunities. Neuroimage.

[71] Burgess A., Gruzelier J. (1993). Individual reliability of amplitude distribution in topographical mapping of EEG. Electroencephalogr. Clin. Neurophysiol..

